# Old Physic and Young Physic; in School and out of School

**Published:** 1848-07

**Authors:** Wm. Treat


### BUFFALO MEDICAL JOURNAL

AND

### MONTHLY REVIEW.

VOL. 4. JULY, 1848. NO. 2.

### ORIGINAL COMMUNICATIONS.

ART. I.—*Old Physic and Young Physic; in School and out of School.* By WM. TREAT, M. D.

(*Concluded, from Page* 17.)

But we hasten to times near our own, and present in a disjoined way, links to a *New School,* the elements of which, as gathered, warrant for it the name of the *Imaginative School,* as, through the imagination, when strictly adhered to, their agents appear to act.

The celebrated Kenelm Digby, a man of rank and of refined education, leads the van. This English knight was at different periods of his life, an admiral, a theologion, a critic, a metaphysician, a politician, and a disciple of alchemy. During his travels on the continent, he became initiated into the mysterious chemical philosophy of the age, and on his return, published an account of the virtues of the sympathetic powder, the knowledge of which he had obtained from a Monk on the continent.

The SYMPATHETIC POWDER was first tried in England, by a Mr. J. Howel, who had been wounded in a duel. The knight, Digby, was in attendance four days after the wound, and dipped one of Mr. Howel's garters in a solution of the powder, unknown to the patient, who was instantly relieved of the pain, and returned home, leaving the garter to be hung up to dry in the chamber of Sir Kenelmn. Soon Mr. Howel sent his servant in great haste, to tell him that his wounds were paining him excessively; the garter was replaced in the solution of the powder, and the patient recovered from his wounds after five or six days of its continued immersion.

King James I., his son Charles I., the Duke of Buckingham then Prime Minister, and all the principal personages of the time were cognizant of the fact. The King being curious to know the secret of this remedy,

Sir Kenelm who revealed it to him; and his majesty succeeded in a surprising manner with it. The Kings's physician, Dr. Mayenne, was made master of the secret, and carried it to France, where a knowledge of its composition was obtained, not however, until the secret had been repeatedly sold at an enormous price, to distinguished individuals, who tested its wonder-working powers. Testimonials of kings, princes, dukes, knights, and doctors, were not wanting. But the powder proved to be nothing but crystalized blue vitriol in an astrologico-chemical way pounded and mixed. This sympathetic powder was said to have the faculty, if applied to the blood stained garment of a wounded person, to cure his injuries, even though he were at a great distance at the time !

A congener if not contemporaneous, was the famous Weapon Ointment, the wonderful cures of which are attested to by Fabricius, known as Hildanus, a learned surgeon, perhaps the most so of his time, as also by Lord Bacon. This ointment, we ought rather to say these ointments, for there were a variety of receipts known under the general name—Hoplochrisma, ('*Oplon*, weapon; *chrisma*, salve,) was used for the healing of wounds, but, instead of being applied to them, the injured part was washed and bandaged, and the weapon or a wooden imitation of it, where the real one could not be had, was carefully anointed with the unguent. Portions of mummy, of human blood, and of moss from the skull of a thief hung in chains, or other substance which addressed the imagination, were favored ingredients. Hildnaus could not resist the cry of *facts* and the solemn assertions respecting its efficacy, though tempted to believe that its efficacy consisted in the washing and bandaging the wound, and then letting it alone. Treating of the attested facts, thus he reasons: "As the ointment is applied to the weapon and cannot reach the wound, it follows of necessity that the devil must have a hand in it;" and though he failed in an experiment with the ointment in the case of a lady, who had received a moderate wound, he accounts for it by the devout character of the lady, over whom the devil could have no control!

Lord Bacon says in his Nat. Hist. in so many words, "as yet I am not inclined to believe in it." His remarks, however, upon the asserted facts, show a mixture of wise suspicion and partial belief; but he adds, in conclusion: "Lastly, it will cure a beast as well as a man, which I like best of all the rest, because it subjecteth the matter to an easy trial."

That wizzard of the North, in the third Canto, 22d stanza, of the Lay of the Last Minstrel, alludes to this kind of surgery, in practice by women,

'tis true, as in medicine it appears to be their peculiar province to tread the footsteps of physicians, bearing the urn of tradition, in which is embalmed the ancient and honorable of what was once held nearest and dearest to heart. The stanza reads thus:—

> "But she has ta'en the broken lance,
> And wash'd it from the clotted gore,
> And salved the splinter o'er and o'er.
> William of Deloraine, in trance,
> Whene'er she turned it round and round,
> Twisted as if she gall'd his wound."

Beaumont and Fletcher, Massinger and others, also allude to this. In the "Witch of Edmonton" (iii. sc. 2) Frank says, after the stabbing of his mistress:—

> "This follows now
> To heal her wounds by dressing of the weapon."

The poet-laureat and historiographer to Charles II,—Dryden—who lived in these magical times, alludes to this ointment more than once in his Tempest. Thus:—

> *Ariel*—"When I was chidden by my mighty lord,
> For my neglect of young Hippolito,
> I went to view his body, and soon found
> His soul was but retired, not sallied out:
> Then I collected
> The best of simples underneath the moon,
> The best of balms, and to the wound applied
> The healing juice of vulnerary herbs.
> His only danger was his loss of blood:
> But now he has waked, my lord, and just this hour
> He must be dressed again, as I have done it.
> *Anoint the sword which pierced him, with the weapon salve, and wrap it close from air, till I have time to visit him again.*
> *Miranda* enters unwrapping the sword.
> *Hippolito* exclaims, O, my wounds pain me!
> *Miranda.*—I am come to ease you.
> (She wipes and anoints the sword)
> Does it still grieve you?
> *Hippolito*—Now methinks there's something
> Laid just upon it.
> *Miranda.*—Do you find no ease?
> *Hippolito*—Yes, yes, upon the sudden all this pain
> Is leaving me—sweet heaven, how I am eased."—*Act.* v. *s.* 2.

But we are admonished we must leap the ages past and come to the present century. For

> "At every step,
> Solemn and slow the shadows blacker fall."

*Cleon*, in Massinger's play of the Emperor of the East, (1585–1639) must assist us across the gulf of time, with his parchment-reading, else our leap were too great for our strength.

> [Enter *Cleon*, reading the Doctor's parchment, the Doctor waits without.]
> "The triumphs of an artisan
> O'er all infirmities, made authentical
> With the names of princes, kings and emperors,
> That were his patients.
> ——— He swears, within three days
> He'll grub up your gout by the roots, and make you able
> To march ten leagues a day in complete armor."—iv. 4.

We verily should have believed this were the special herald of the Metallic Tractors, first boon of this century, were there not so many Magi to be proclaimed ere the sands of the first half of the century were numbered.

In 1796, from that slandered State which is said to manufacture much that is wooden, there was put forth by one Dr. Elisha Perkins, of Norwich, two metallic cones, one apparently of brass the other of iron, sum total in value, a York shilling, but by account of their peculiar virtues, as a special favor, sold to clergymen and some others for *five* guineas. Our trumpeter Cleon has "opened the ball" with reference to the efficacy of something, perhaps of these. In 1798 they have crossed the ocean to Great Britain, and the printing press there, as was the case here, has marshaled its forces in squares and columns. to greet and commend this great discoverer of the age. Aye, these Tractors are already employed in the royal hospital in Copenhagen. The Danish physicians have published of their success in a respectable octavo volume. In 1804 the Perkinean Institute is founded in London, and the Perkinean Society has public dinners at the Crown and Anchor—a poet-laureate who had invested capital for one-fifth of the patent, will soon write his "Terrible Tractoration," proving the excitement of his mind upon the subject, if naught beside.

Dr. Fuller, of London, advised every family to keep the Tractors in their houses, as a cure-all, "without the expense or danger attendant upon common medical practice." The Royal College of Physicians has accepted a pair of the Tractors, also a copy of the book of facts, voted thanks, and laid them upon the shelf without troubling themselves to investigate their properties. But why heed that?—the Perkinean Institute is founded, Lord Rivers is elected President, and eleven worthies Vice Presidents, among whom is Governor, son of Dr. Franklin.

Lord Henniker, a member of the Royal Society, who bears the reputation of a man of judgment and talents, has condescended to patronize, and has bought three pairs of the Tractors. Besides the Chaplain of the Earl of Dunmore, also of the Prince of Wales have commended them. What matters it then, that of American certificates, among the honorable, we find 34 physicians and surgeons, thirteen clergymen, most of whom are doctors of divinity, one professor of natural philosophy in a college, and two members of congress. The mass of Facts as to cures wrought, are before us, hear the report of the Perkinean Committee:—

"The cases published [in Great Britain] amounted, in March last, to about 5000; suppose only one in every 300 of cases have been published, (and the proportion is probably much greater) it will be seen that the number to March last, will have exceeded 1,500,000!"

We trust with this amount of attested cures, and all of them belong to the class of FACTS with which medical literature and street talk abounds, no one of my auditors will be found skeptical. Were it thought probable we would summon,(as was done by the Perkinean Institute)"the injured ghosts of Harvey, Galileo and Copernicus to shame them." True the Baillies and Heberdeens—men whose names have come down as synonymous with honor and wisdom, bore reproaches and left them to their fate, as has been done with Magnetism and Homœopathy of the present day. But some others, believing the whole to be a delusion, essayed a test. Dr. Haygarth, of Bath, tried tractors of his own invention on patients with different complaints, and they operated as well as the five guinea ones, so long as the patients believed them to be genuine. His were made of lead, wood, nails, pieces of bone, slate pencil, and even stems of tobacco pipes. Dr. Alderson employed sham tractors, made of wood, and produced such effects on five patients, that they returned solemn thanks in church for their cure. Ann Hill's case may serve as a type of the whole:—She had suffered for some months from pain in the right arm and shoulder. The (wooden) tractors were applied in the usual way, by drawing them over the afflicted part very lightly. In the space of five minutes she expressed herself relieved in the following apostrophe: "Bless me! why, who would have thought it, that them little things could pull the pain from one? Well, to be sure, the longer one lives, the more one sees." As many now urge of other fancies if one gets cured, what matters it how? Their poet-laureat so sang:—

"What though the *causes* may not be explained,  
Since these *effects* are duly ascertained,  
Let not self-interest, prejudice and pride,  
Induce mankind to set the means aside."

These magnetic tractors though wonder-working (composed of whatever inorganic substance from brass to a pipe stem) were wanting in one class of phenomena to ally them with mesmerism, somnambulism and clairvoyance—it was reserved for the digital points of the human hand or that spherical orb, the leering eye, to accomplish that spell which Massinger's counterfeit Roman Actor had previously enacted: Hear him:

*Æsopus.*—Is he not dead?
*Paris.*—Long since to all good actions.
You may with safety prick him,
Or under his nails stick needles, yet he starts not.
———We must use
Some means to rouse the sleeping faculties
Of his mind, there lies the lethargy. Take a trumpet,
And blow it into his ears; 'tis to no purpose,
The roaring noise of thunder cannot wake him
And yet despair not; I have one trick left yet.
I will cause a fearful dream
To steal into his fancy, and disturb it
With the horror it brings with it, and so free
His bodily organs.
*Domitiames Cæsar.*—'Tis a cunning fellow,
If he were indeed a doctor, as the play says;
He should be sworn my servant, govern my slumbers,
And minister to me waking."—*Act. ii, Scene* 1.

The chief difference between the poet's player two centuries ago, and the public believer at this time is, the first substitutes an "*if* he were indeed a doctor as the play says"—and the latter makes him one, by mere force of the will, and then we have enacted as may be read in Deleuze's book, which we are told is best authority on magnetism—of an eye destroyed by small-pox, restored; (p. 149) of a leg six inches short and a callosity as big as the fist, in six weeks the callosity is reduced one half and the leg made three inches longer, facts of course, and well vouched for! (p. 153)

But here we must enter up our protest to farther exhibitions as recorded page 251, of souls making long journies of knight errantry and leaving their bodies behind, apprehensive as we are, that as fared it in an instance cited by Pliny, it may happen to others,—the ghost went out, and the body being destroyed before its return it could not get a habitation, a loss not made up to science until this our day, and bearing a caution with it, as without care much loss might again accrue to science! For many wonders we trust have yet to be revealed, and useful ends attained, besides those related of trees mesmerised, whereto lady visitors in the garden were attracted and spell-bound. (*p.* 230-31) Orchards might thus be protected from robbery.

In mechanic arts it would be useful to reveal disordered mechanism—in trade, quality and fitting prices of sugar and tea—prices current abroad, or prospective crops, and then the mere trifles of health and ministry of potent medicine will be left perhaps to those triflers whose time and thoughts tend to nothing else! Or, relying upon present revelations by Deleuze, a pitcher of magnetised water shall cure all diseases. Crudities, as well as wheat bread, and water shall be given to crying children who while under the operation shall declare as do the adults he names, the one is cake, the other wine or whatsoever is willed as it is willed by the magnetiser and the pitcher of precious fluid. (*p.* 355.) But alas! while we somnambulise

> "Dear lost delusion! Truth's too fervent ray,
> Strikes thy bright frost-work, and it melts away."

Not however without leaving its fantastic fret-work to be interwoven with real science for private fraud and peculation..

Of HYDROPATHY we have time but for one or two remarks. If no benefit is here derived from the journey to a special place, often invigorated with mountain air, regular habits, gentle exercise and attendant diet, then must be attributed cures to the means which Cardon teaches thus: "'Tis opinion which makes or mars physicians; and he doth the most cures, (according to Hippocrates) whom most trust." Cardon has claims to be heard on this subject, for he lived centuries anterior to our time and Burton in his Anatomy of Melancholy, says: "Cardon cracks that he can cure all diseases with water alone as Hippocrates did most diseases with Hellebore alone." (p. 439.) This verifies that old adage:

> "There is nothing so new as that which is forgot."

In keeping with Culpepper's Herbal we have that of William Salmon, printed about 1690, titled New London Dispensatory, the plants enumerated have virtues similar. We take it up now for the purpose of citing a few animal remedies. Here we read again of the moss from a dead man's skull as an ingredient to a magnetic powder, (p. 77); of amulets hung about the neck or the ears, the elk's hoof for instance inclosed in a ring, or beetle-bugs; of hair and nails from the patient cut fine and put in a hole bored in an oak or plum tree and plugged in, or, given to birds in a roasted egg—or mixed with wax and stuck to a live crab, perhaps the soldier crab, casting the animal into the river again, curing consumption, (p. 190); of artificial mummy, when real is not to be had, made from the carcass of a young man, (some say red-haired) not dying of a disease, but killed; mixed with aloes, myrrh, and spirits of turpentine and dried in a shady place; this dissolves blood, helps coughs and cures wounds. Spirit of man's brains—

Lamb's grease with jelly of vipers, (p.197)—Chameleon's liver to dissolve love—Ram's brains and honey to cause children to breed their teeth easily, (p.198)—or dog's teeth calcined and so mixed; of oil of bricks; a quintessence, an ingredient of which is the left hind foot of an elk, rasped. (p.509.)

Of the *aqua divina*, toe divine water, made thus: The whole carcass of a man violently killed, cut in pieces and distilled twice or thrice in a retort. It is reputed to have a magnetic power. We are gravely told, if to a teaspoonful of this water, you put a few drops of the blood of a sick person, and set them on the fire and they mix, the sick recovers, if not, the sick dies for want of blood, (p.195.)

If authority were wanting confirmatory of the prevalence of such practice and its test by centuries of experience, (and men of more note) we might have selected the most of these from an old London Dispensatory 1618; or from John James Manget's ponderous Latin folio, 1703. The latter work, aside from this, is really of much science for its day.

These treat of oil from swallows, worms, millipedes, frogs, scorpions, &c. Let two recipes suffice:

℞. Vipers, living, large and fat, No. iii; Spanish wine, ℥ii; St. John's Wort ℥viii—express and evaporate. It cures gout, leprosy and other diseases of the skin. The preparation to be made if possible when the sun enters the sign Scorpio or Cancer. (Manget, p. 463, also p. 481–2.)

For tooth-ache, put powdered worms into the cavity.

Here we are reminded we have quite overlooked another out-of-school practice which has held rule with the multitude for centuries and has strong affinities with the decidedly foremost of our own time. We allude to the doctrine of SIGNATURES, applicable to mineral, vegetable and animal remedies. This mode sets out with resemblances of the medicaments, fancied or real, acting, when taken, as curative to the parts which they are supposed to resemble—for instance, if a leaf be heart or liver shaped it shall cure the like. "Walnuts are said to have the perfect signature of the head; the outer husk or green covering represents the pericranium, or outer skin of the skull, whereon the hair groweth, this is good for wounds of the head. The woody shell has the signature of the skull, the little yellow skin or peal, that of the dura or pia nater, which are the thin scarfs which envelope the brain. The kernel has the very figure of the brain, and therefore is profitable for the brain."

Of animal medical agents: The stronger the animal the better the remedy. Extract of man's skull cures most of the diseases of the head.

A quintessence made from man's flesh is a universal remedy, S. 407, Heart of a Hyena helps palpitation of the heart; Lion's flesh helps the palsy—and every part of this animal as also of the Wolf is of efficacy in the diseases of like parts of man. Powdered worms taken expel worms—this remedy coming down by tradition we have met in actual practice among old lady traditionalists in this city! Dog's liver, especially that of a mad dog, dried, powdered, and taken, cures the bite of a mad dog. (p.202) An ointment of hair-ashes, distilled with honey makes hair grow, S. 189: for "The Linimentum Ischiadiam"—hip liniment, we read from Manget p.176, ℞. Canes novissime natos, et Talpas viventes, cum Lumbricores terrest, etc.; that is, take new born puppies and living moles, and earth worms—add red wine and goose grease, make a liniment to rub the parts. While Salmon says: The heart of an Ape, a teaspoonful, dried and drank in powder strengthens the heart and increases boldness—a mode by which we almost imperceptibly approach—

Homœopathy.—The hint of the similarity of this doctrine to that of signatures, we have from Menzel's history of German literature, Menzel himself being a *dillettante* of the Homœopathic school. Our friends of this school certainly will not object to our thus approaching the subject hand in hand with one so distinguished among the German literati, with Manget a German king's physician, bearing some "similia" in fore-front, when they themselves boast for its modern phase, German extraction—the founder Saml Hahnemann,—and of modern German princes as its especial patrons.

We under these advantages however, approach this to us unsolved riddle with not a little caution. Here we are conversing of dogmas held by our neighbors, among whom we number many of our best friends, whose honest opinions we would treat kindly if for no other reason, for their kindness manifested towards us, how absurd soever the dogmas to which they tenaciously cleave may appear. To treat the subject gravely we shall find much difficulty, but as it has fallen into the meshes of our discourse, at this point, we must needs with our lance run a tilt with it, as our time will not suffice, at this hour, to venture upon a minute dissection.

We have called Homœopathy an unsolved riddle and we do it notwithout reflection:—for as taught by Hahnemann it affirms that an infinitessimal quantity of matter is more potent to kill and more potent to heal than its greater:—that this matter thus rendered potent, cures or kills by its similitude, acting as cause or effect, *similia similibus curantur*, that which causes, or its like, cures, is the pass-word, the *open seseme* to this new school. Once entered if we follow its founder we have soon started another hypothesis,

(the discovery of either one of which, if true, would be fame enough for any one man) that at least seven-eights of all chronic diseases originate from itch.

Though in the outset we resolved to be grave, we confess one does feel a slight infinitessimal tickling at the mid-riff, by operation of so potent an agency as that strong infinitessimal, *mental conception of its causative*, but with this *fact*, this experience alone tending to our conversion, at present we should be unwilling to encounter the fully engendered disease without privilege of actual scratching—for such a disease when really present is not to be laughed off! One other feature in this riddle, though not laid down by Hahnemann, to be observed in all their *materia medica* is this, that these *similia*, causing and curing disease, each have an antidote affixed, which is not the first similia, but is pressed into service to counteract the first similia. Obviously the reception of such antidotes is a denial in practice of the Hahnemannic theory! These *similia* are cured by *contraria*, or the word antidote in this connection conveys no meaning to us. But we do not press this, each of these doctrines have been set aside by schismatics who rank as Homœopathists. But, this was the Homœopathy of Hahnemann, and such it was a few years ago, when curiosity led us to an examination of its tenets. Recently we have re-examined it, and it is but fair that we give its newly acquired features.

The itch dogma, though Hahnemann declares it was a labor of 12 years to innoculate it in, has by his professed followers and leaders too, been pretty effectually scratched out. The German section of the faithful have gone farther and insist upon a little more potency, not infinitessimal, but Eclectic potency, for acute diseases, as also do many Americans. Another phalanx march forth, and a pretty large majority this, with banner unfurled, repudiating that part of the original doctrine which affirms *similia similibus curantur*, in so far as it teaches that the medical cause may by improper or unskilfull use beget a new, yet similar disease,—the excess of potency first killing out, as originally taught, the little whelp of a disease and substituting the potent roaring lion, uncaged like the seven plagues from the unsealed apocryphal vial—unconfined from the sugar-coat of mail!

Do we here mistake? Let us quote from the Organon, there we read "Homœopathy cures, by opposing to the natural disease an artificial one as similar to it as possible, and sufficiently intense to extinguish it by its predominance. If the factitious disease were weaker than the natural it would remove the latter only in part; if it were stronger, it would no doubt cause it to disappear altogether, but it would leave in its place an artificial disease extremely similar as to symptoms." These doses to be so nicely adjusted let it be remembered are little pellets moistened in the mingled waves of one million lakes two miles in circumference, in which has been blended one drop of Tincture of Chamomile as is given the dose IVth degree Jahr's Manual—though Hull in his translation has omitted to state this dose—other doses there are which are given in the Xth or even higher degrees of attenuation! The difference between the weapon ointment and one of these dilutions of medicine is said to consist in this: "The one is an infinite dilution of the common distance at which it is applied—the other an infinite dilution of the common dose, in which the remedies are given."

It will be observed that the crowning *similia* in the riddle of this school with its three several divisions is:—That conjoinedly they affirm *all*, and singly they deny *all* the teachings of Hahnemann, thus by their individualities, they dig and undermine Homœopathy to its very foundation and chief corner stones!

We designed to speak of the mode of conducting the boasted experiments of Hahnemann,—of the almost utter impossibility of making an attenuated dose which even in the 10th degree of potency consists of the 100th part of the millionth, of the millionth, (this numeral ten times repeated,) of a grain of an oyster shell. Let this one instance suffice of his mode of conducting his boasted experiments copied from the 213th page of Hahnemann's Treatise on Chronic Diseases:—"After dinner, disposition to sleep; the patient winks." Nine days after taking the remedy, again he adds: "After dinner, prostration and feeling of weakness."

The remedy was oyster shell prescribed in fractions of the sextillionth or decillionth degree. The action of a single dose of the size mentioned, according to Hahnemann, does not fully display itself in some cases, until 24 or even 30 days after it is taken, and in such instances has not exhausted its good effects until towards the fortieth or fiftieth day—before which time it would be absurd and injurious to administer a new remedy!

These doses we are also informed may be rendered inoperative by the presence in a room of aroma, even Cologne water. Gunther in his treatise on diseases of horses adds that homœopathic treated horses should not even stand beside horses that have taken allopathic medicine—though cologne is so antagonistical—the perfumery of a stable is not suggested as an inconvenience! Yet chemically this, as also the secretions of man, or animal, would neutralize most of *such* doses before they could even reach the stomach.

But of Homœopathy this must suffice, when we add there is not in our knowledge a single practicing physician who is of this school! We say it fearlessly, let Hahnemann be judge and exponent to him who affirms the contrary! When we hear of sensible effects from reputed homœopathic doses it will be well to bear this in mind—elsewhere we cannot answer for them.

In the school which we have ventured to name the Imaginative, we trace germs of the *archeus* of Van Helmont, as also of the *anima* of Stahl, which we cannot now stop to define; and why need we? Chaucer, the father of English poetry, long since wrote

> "Men, by their nature, love new-fangledness
> As do the birds that men in cages feed."

To batter down one set only makes way for another; therefore retaining our equanimity, we can only say of these, in language of Chapman:

> "——— this wind, that doth ring so in your ears,
> I know is no disease bred in yourself,
> But whispered in by others, who in swelling
> Your veins with empty hopes of much, yet able
> To perform nothing, are like shallow streams,
> That make themselves so many heavens to sight,
> Since you may see in them the moon and stars,
> The blue space of the air,—(as far from us,
> To our weak senses,) in those shallow streams,
> As if thay were as deep as heaven is high;
> Yet, with your middle finger only sound them,
> And you shall pierce them to the very earth."—*Byron's Conspiracy.*

Sir Walter Raleigh having witnessed a quarrel from his study window, which took place in the court-yard below, was so disconcerted at the conflicting testimony of other observers, is said to have burned the manuscript of a work which he had been engaged in writing, containing his observations made on the various countries in which he had travelled, doubting the accuracy of his own eyes:—A process similar to this no doubt has tempted many first to falter, then to fall from belief in remedia lagency of medicine.

Pliny tells us that in Rome physicians were banished for six centuries—and it may be gathered from Livy with what effect when he tells us, to stay the ravages of the plague the Syballine leaves were consulted—the public games instituted—the god Æsculapius in form of a serpent imported, and finally the plague is arrested by Augustus Cæsar's driving a nail! Livy himself expresses no doubt of their efficacy. Had we not the history of such an attempt centuries before—and were we so mentally constituted as a Raleigh, we know of no doctrine so attractive as that of the infinitesimal dose.

Often are we puzzled to know what to receive on testimony and now, while reflecting on the law of evidence, we were moved to refer to the printed Report of the trial of witches, 10th of March 1664, before the

> "Immortal Hale,—for deep discernment praised,
> And sound integrity—not more than famed
> For sanctity of manners undefiled."

To this the writer of the preface adds: "the trial was held before a judge, who for his integrity, learning and law, hardly any age, either before or since, could parallel,—who not only took a great deal of pains, and spent much time in this trial himself, but had the assistance and opinions of several other very eminent and learned persons." The evidence is taken and summed up:—the judge charged the jury

"That there were such creatures as witches, I make no doubt at all! For 1st, The *scriptures* hath affirmed so much. 2d, The *wisdom* of all nations had provided laws against such persons."

How could a jury do otherwise than find a verdict of guilty, when revelation and reason and the judge were agreed! We read in connection, "Dorothy Durent had been on crutches three years, but when the jury brought in the verdict of guilty against Amy Duny, now a convicted witch, to the great admiration of all present, she was restored to the use of her limbs, and went home from the court without crutches!"

The next morning those afflicted came to the Lord Chief Baron Hale's lodging, all spake perfectly, and in good health, though dumb before! Testimony upon medical subjects and reputed cures after this may well confound the ignorant when published in pamphlets or newspapers.

The judge might have saved his reputation here, perhaps, had he been previously in possession of a recipe current in his time. As it may be of service to other judges of law, who prate upon medicine, we help them to it.

Bartholomus Carrichters, in his *Secret*, b. 2. c. 12, published a recipe which is mightily commended by Hector Schlands, in an epistle to his learned friend Gregorius Horstius; see Horstii Epist. Medic i, §7. 1612. "℞. Dogs' grease well dissolved and cleansed ℥ iv; Bears' grease ℥ viii; Capons' grease ℥xxiv; three trunks of the missletoe of hazle, while green, cut it in pieces and pound it small till it becomes moist; bruise it together and mix all in a phial. After you have exposed it to the sun for *nine* weeks, you shall extract a green ointment wherewith if you anoint the bodies of the *bewitched*, especially the *parts most affected*, and the joints, they will certainly be cured." This recipe was tried with amazing success in the case of a young girl whose condition was truly deplorable; for she vomited feathers, bundles of straw, and a row of pins stuck in blue paper, as fresh and new as any in the pedlars stall, pieces of glass windows, and nails of a cart wheel; as may be seen in the "Wonderful and True Relation of the bewitching a young girl in Ireland, 1669. by Daniel Higgs.

The hearer by this time no doubt has asked wither does all this tend—our answer is, to this conclusion. We feel with you that we have already exceeded the limits of one lecture—but find one apology therefor, and is this not enough? We have been treating upon a subject that ere to morrow's sun, may, with you, involve the question of life or death! For hecatombs of human beings are now being sacrificed to some of these Molochs! Our aim will have been attained, if you shall have perceived from what has been adduced: That the Old or Eclectic school has ever been open to, readily received, and adopted the principles of philosophy, and of the sciences as they have advanced in every age, and rejected their errors in like ratio. That all ages preceding have had their crudities and wondrous medical cures and the 18th and 19th centuries are not wanting in theirs. That testimony *ab extra*, that is from without the medical profession, and that too volunteered by the most gifted in their time, of the efficacy of panaceas, was as plentiful as now; that is most likely to be of worth which emanates from individuals who cultivate one department of learning and in giving testimony confine themselves there; that kings, princes, bishops, priests, judges and the *vox Dei* of a populace may utter a true voice in many matters, yet without knowledge of collateral sciences are illy qualified to recommend, much less to judge of new views in medicine, or weigh the old, be they superficial or profound. That in short a King James, Berkley, Bacon, Wesley or Milton, or among moderns Bulwer, Martineau or Bryant, and those seated in high places may be good rulers, theologians, philosophers, novelists or poets, yet poor physicians, mechanicians, navigators, or land surveyors. The world is too wide, mechanism too complicated, medicines too numerous in kind, and the laws which govern their action too dependent on time, and occasion and circumstance, for any, even the many-sided, and most gifted, and most studious to hope to compass them all.

All of the articles of medicine, and most of the doctrines of the schools of which we have spoken have had their centuries of proof of their value. Many of them are now discarded as ridiculous, though few indeed were there of them which were permitted to die without benefit of clergy!

Homœopathy, Hydropathy, Mesmerism in medicine, (we say nothing of the psycological phenomena,) cry loudly for their adoption, and their admirers rest their *faith* in their *experience.* But such experience without collateral information to combine and contrast, often *has* and *may* lead to results like the following, copied from Burton, as illustrative as well as characteristic.

"Last of all it is required that the patient be not too bold to practice upon himself, or try if he read a receipt in a book. (Newspapers were not in his time.) That which is well for one man may not be for another. An ass and a mule went laden over a brook, the one with wool the other with salt; the mule's pack was wet by chance, and the salt melted, his burden the lighter, and he thereby much eased; he told the ass, who, thinking to be as well benefitted, wet his pack in the next water, but it was much heavier, and what become of him my story saith not; look about and he may yet be found, *perhaps!*"—*p.* 305.

Thus are we to distrust the power or competency of observers, who boast even of experience when setters forth of strange doctrines, which war with all philosophy and science, which are parts component of the science of medicine. In the words of the celebrated Mr. Locke, from his Essay on the Human Understanding, we add:—"Those who have not thoroughly examined to the bottom all their own tenets, must confess they are unfit to prescribe to others: and are unreasonable in imposing that as truth on other men's belief which they themselves have not searched into, nor weighed the arguments of probability on which they should receive or reject it."

The field of science is one of battle no less than one of progress. Errors are to be warred against; and though the veterans in medicine are not often knighted for their courage, yet there are not wanting Wellingtons and Napoleons, in their ranks, though unknown to fame. Those who have faced and stayed the march of small-pox, plague, cholera, and ship-fever, are its true heroes, battling manfully, and the past year's experience tells of their fall, unappreciated, scarcely known a few leagues from that field of chosen labor,

> "Where the equal thought he bore of life or death,
> Made him faint on neither side."

> "And where he hath used no ambition to commend his deeds,
> The *deeds* themselves, though mute, spoke loud the doer."

"The wages of every noble work do lie in heaven, or nowhere," says Carlisle, and thither have they been borne to their reward.

To those medical students from the University who have given an hearing, we have but to remind them, in conclusion, of that law in optics, whose sight through a pin-hole, presents objects greatly magnified, by reason of a few rays of light and a narrowed vision—here he may find the new visionists. That other law of optics, of known worth to the astronomer, presents to you—he sees farthest and calls out of the vast expanse the most stars, who, having a single eye fixed upon an object as a pole-star, causes the other to scan the firmament obliquely to the compassing of the heavens. The Eclectic thus in the pursuit of knowledge, with an eye fixed upon the keen piercing demands of suffering humanity—there should find his pole-star, while he looks out with the other into the chaos of the past, and resolves the nebulæ of opinion of the present, which tend to distract and darken the uderstanding of the superficial. To the light of inspiration he lays no claim—blind chance has added but little—but to the light of reason he must ever look; and not with both eyes open or shut, be wiled away into fens or morasses by the *igniis fatui* of ephemeral lights which surround him.

Yet when you shall go out into the practice of medicine, be it in city or country, you may from the countenance given to quacks, run the hazard depicted by the poet. Sufflce it to say, judging from the past, some there be who will not escape:—

> "You cannot be too cautious, nice or dainty
> In your society here, especially
> When you come raw from the University—
> Before the world has hardened you a little;
> For as a buttered loaf is a scholar's *breakfast* there,
> So a poach'd scholar is a cheater's *dinner* here:
> I ha' known seven of them *supp'd* at one meal."

---

NOTE.—For facts and phraseology, not however without refering to original sources, when treating of sympathetic powder, weapon ointment, metalic tractors, tar-water, and Homœopathy, the writer is often indebted to the author of two published lectures—Dr. Holmes of Boston, entitled "Homœopathy and its kindred delusions," a small volume of interest to the curious in such subjects.

The reader will please notice the following among other ERRATA:—Page 4, for cerro-strati, irotheral, isochemical, read *cirro strati, isotheral, isochimenal;* page 5 line 30, *ridicule,* for ridiculous: page 6, line 3, *physical,* for physic: p. 11, line 25, *pages* for papers,—line 27, *large,* for red-hot: [no such exaggeration is needed!] p. 14, Buffon *iv,* 210, for 18, 210: p. 17, line 9, *Tajura,* Egypt, for Tassura.

